# Chinese herb related molecules of cancer-cell-apoptosis: a minireview of progress between Kanglaite injection and related genes

**DOI:** 10.1186/1756-9966-27-31

**Published:** 2008-08-21

**Authors:** Yun Lu, Chang-Sheng Li, Qian Dong

**Affiliations:** 1 Department of Hepatobiliary Surgery, Affiliated Hospital of Medical College, Qingdao University, No.16 Jiangsu Rd, Qingdao 266003, PR China; 2 Center of Cell and Molecular Pathology, Affiliated Hospital of Medical College, Qingdao University, No.16 Jiangsu Rd, Qingdao 266003, PR China; 3 Department of Pediatric Surgery, Affiliated Hospital of Medical College, Qingdao University, No.16 Jiangsu Rd, Qingdao 266003, PR China

## Abstract

Many kinds of Chinese herb had been confirmed to have the character of anti-tumor, clinical reports about anti-tumor effects of Chinese herb had also been found in recent years, but most of the reports were focused on the clinical treatment of effectiveness for Chinese herb, on the other hand, review about Chinese herbal related with molecules on cancer-cell-apoptosis was seldom, many scientists could not believe such kinds of clinical describes about anti-tumor effects for Chinese herb, because these describes were lack of molecular biology evidence. Kanglaite(KLT) injection is an anti-tumor new drug which extracts from Chinese medicine-*coix seed *with modern advanced pharmaceutical technology, it is also a new biphase extended-spectrum anticancer medicine, the food and drug administration(FDA) of United States also approved a phase II trial of KLT to test its efficacy in treating non-small-cell lung cancer. Some studies show it could inhibit some anti-apoptotic gene and activate some pro-apoptotic gene, its injection solution is one of the new anticancer medicine that can significantly inhibit a various kinds of tumor cells, so it has become the core of research that how to further explore KLT injection to promote tumor cell apoptosis by impacting on related genes. In this review, the relationship between KLT and some tumor cell apoptosis molecules had been discussed and reviewed generally.

## Review

In recent years, with the lucubrate on tumor cell biology and molecular biology, it has been recognized that the occurrence and development of tumor is not only the result of cell proliferation disorders and disdifferentiation, but also closely correlated with the abnormal apoptosis [1,2]. Although abnormal apoptosis can promote the occurrence and development of tumor, we can also treat tumor by promoting apoptosis of cancer cell [3,4]. Thus it has become the new target in oncotherapy by way of inducing apoptosis of cancer cell. Kanglaite(KLT) injectionis a diphasic broad-spectrum anti-tumor new drug which has depressant effect on many kinds of tumor cells, it is extracted from the chinese crude drug-coixenolide [5,6] and made use of the latest and most complex modern high technologies in process of preparation [7]. Animal experiments show that KLT mainly block G2+M phase of cell circle, thereby reducing the mitotic division of cells, so the proliferation of tumor cell was inhibited, at the same time it can also activate some pro-apoptotic factor, and further lead to apoptosis [8]. Clinical application also shows that combined with chemotherapy, KLT has a good effect on the treatment of advanced cancer, particularly in digestive tract cancer, for example, the patient's life span and quality of life improve significantly. The finding show that this preparation has significantly depressant effect and preonunced curative effect on a variety of cancer cells [9]. Although the therapeutic measure of liver cancer contain surgery, radiotherapy, chemotherapy, interventional therapy and so on, their effect is not satisfactory so far [10,11]. However, with the development of cell biology, the theory of apoptosis presents a new hope and path for the treatment of liver cancer, The present study has found that KLT plays an important role in promoting apoptosis of hepatoma cells [12]. It has been known that the apoptosis of hepatoma cells is triggered by a variety of receptor-mediated cell signaling, and a variety of protease take part in the apoptotic signal transduction. In addition many kinds of genes are also involved with apoptotic regulation of hepatoma cells. In this paper, the relationship between KLT and the hepatoma cell apoptosis molecules is going to be discussed and reviewed generally.

### The influence of KLT on p53

There are two types of p53 genes: the wild type p53 gene and the mutant p53 gene. The wild type p53 gene, which is also known as the guardian of gene, is indispensable to regulate the normal cells circle [13,14]. On the one hand, as the important regulating factor in the process of apoptosis, the wild type p53 monitor the integrity of genes all the time, on the other hand, as the nuclear transcripton, it can respectively combines with DNA and RNA polymerase to regulate expression of gene, it can also inhibit the synthesis of DNA, take part in the repair of DNA, induce cell growth stop at the phase of G0. In addition, the wild type p53 gene can induce the Fas-mediated cell procedural death after the damage of DNA, so that the regular growth of cell is maintained. Wang JJ [8] had found, while discussing the anticancer mechanism of KLT injection, that the labeling index of wild type p53 in treat group which received KLT injection is 16.8%, while the control group had no expression at all. Furthermore, in the experiment about KLT-induced apoptosis, Bao Y [15] discovered that compared with the control group, the mRNA level of the wild type p53 gene significantly Increased in 20 μl/ml KLT experimental group after 48 hours. And in the experiment about apoptosis of multidrug resistance phenotypic human breast cancer cell line MCF7^adr ^and its cell cycle arrest that induced by KLT. According to the immunohistochemical detection, Guo JW [16] found that the wild type p53 gene of MCF7^adr ^in the control group is negative, but the wild type p53 gene of MCF7^adr ^in the KLT experimental group is midrange positive, it can be assumed that KLT could up-regulate the expression of p53 and extend half life of p53 protein. In addition, Wei CY [12] observed 34 cases' hepatoma carcinoma cell that cultivated in vitro and their changes after treated with KLT, the results indicated that, compared with the control group, the apoptosis of hepatoma carcinoma cell in KLT experimental group is very significant, there was significant difference between them, at the same time, the labeling index of wild type p53 in the treat group is (8.39 ± 1.42)%, but the labeling index of wild type p53 in the control group (2.11 ± 0.97)%, there was significant difference between these two groups. the phenomenon recorded here is the same as the studies of Wang JJ, Guo JW and Bao Y which had indicated [8,15,16]. In conclusion, KLT injection's may induce the apoptosis of tumor cell by way of up-regulate the expression of p53 genes.

### The effect of KLT on the bcl-2 genes

Proto-oncogenes bcl-2 is the most definite apoptotic antagonist gene so far. In 1984, It was first cloned in t(14; 18) (q32; q21) chromosome translocation of follicular lymphoma cell line. But many studies had also confirmed that the high expression of this gene might inhibit the apoptosis of a variety of cells as well[17,18], thereby it can participate in the occurrence of a variety of tumor. When studying the anticancer mechanism of KLT, Wang JJ [8] found that the labeling index of bcl-2 gene was (16.80 ± 3.77)% in the control group, which is higher than that (6.6%) in the 10 μl/ml KLT treat group. Moreover, accompanied with the concentration of KLT increase, the gene expression of bcl-2 decreased in KLT group. In the experiment about KLT-induced the apoptosis of pancreatic cancer cells, according to the Western blot analysis, Bao Y [15] discovered that the expression of bcl-2 protein decreased after 72 hours when application of KLT at 20 μl/ml, the results of these two experiments above-mentioned may indicate that KLT induced the apoptosis of cells by down-regulating the expression of bcl-2 genes. Nevertheless, when studying the KLT- induced the apoptosis of cancer cell(HL60), Li Y [19] make use of RT-PCR to detect the the gene expression of bcl-2, there was no significant change in genetic transcription after 24 hours when using KLT at 10 ul/ml. So, whether KLT induces apoptosis of cancer cell by down-regulating the expression of bcl-2 genes isn't yet clear, and its role in hepatoma is not learned, which requires further study and research.

### The effect of KLT on Fas-genes

Fas gene is located on the No.10 chromosome q23, with a length about 25 kb, the codogenic Fas protein consists of 325 amino acids and it can be expressed in many tissues. When Fas protein combining with Fas ligand, signal of apoptosis is send to the cell and the apoptosis of cell is induce [20,21] Many anticancer drugs can induce the apoptosis of cancer cell by up-regulating the expression of Fas gene [22]. when studying the KLT- induced the apoptosis of cancer cell(HL60), Li Y [19] make use of RT-PCR to detect the the gene expression of Fas, she observed that genetic transcription strengthened after 24 hours when using KLT at 10 ul/ml. Han SX [23] proved that KLT injection can induce the apoptosis of human cervical carcinoma cell by raising the level of Fas gene. Similarly, the experiment, which detected the expression of Fas receptor on the surface of osteogenic sarcoma cell under different concentration of KLT [24], showed that under the KLT concentration of 0 ul/ml, 1 ul/ml, 5 ul/ml, 10 ul/ml and 20 ul/ml, the amount of Fas mRNA detected by the RT-PCR analisis is (0.12 ± 0.02) ul/ml, (0.27 ± 0.05) ul/ml, (0.35 ± 0.09) ul/ml, (0.46 ± 0.14) ul/ml and (0.51 ± 0.16) ul/ml respectively, so they considered that accompanied with the concentration of KLT increase, the level of Fas gene in the cancer cell significantly increased. Anyway, KLT may induce apoptosis of cancer cell by up-regulating the expression of Fas genes, but its effect on the Fas gene of liver cancer cell should be further studied.

### The effect of KLT on caspase-3

Caspases is a group of prolease that induce the apoptosis of cell. Under the normal circumstances, the strict substrate specificity and high effectivity of the activated caspases can assures its narrow spectrum of proteolysis during the process of apoptosis [25,26]. The caspases selectively Shear a group of protein in a simpatico way that lead to functional failure or structural changes of the protein. If the activity of caspases is suppressed, the cellular apoptosis could be disturbed, which could lead to the occurrence and development of tumor, because the dynamic balance between cellular apoptosis and proliferation is disturbed. Caspases-3 and Caspases-8 are the most widely studied in caspases family. Caspase-3, as a important member in the caspases family, is a major functional enzyme in the pathway of cellular apoptotic signal[27,28]. Caspases-3 can cause the clearage of its substrate PARP(116 × 103) and transferred into opyeptide 24 × 103 and 89 × 103 and thereby activate the endonuclease to trigger the complete degradation of DNA. Many factors that regulate the cellular apoptosis can react through caspase-3 prolease. The depressor of caspase-3 can restrain the activation of caspase-3 and degrade the activity of it, so that the apoptosis of cancer cell is restrained [29,30]. Bcl-2 and p53 both interact with caspase family [31-33]. Some report have discovered, while studying KLT-induced the apoptosis of human pancreatic cancer Paru-8988 cell, that the increase of caspase-3 total protein also show obviously time dependence [34]. Moreover, through the test on the substrate of caspase-3-PARP, no degradation product (89 × 103) strap was found in the control group, but the degradation product (89 × 103) strap was discovered after 6 hours in KLT group, which indicates that caspase-3 have enzyme activity. However, no study or research about the effect of KLT on the liver cancer cell had done.

### The effect of KLT on other related genes

PCNA (proliferating cell nuclear antigen) is a subunit of DNA polymerase and cell cycle-dependent protein whose maximum appearance in the S phase. Some research shows that the expression of PCNA is connection with the low grade tissues[35,36]. Wang JJ found that the gene expression of nuclear PCNA increased obviously, and the labeling index of PCNA was 15. 2% after 48 hours when 0.2 mg/ml KLT effected on the renal cancer cells, while hardly any gene expression of nuclear PCNA was observed in the control group [8]. They believed that PCNA took part in the reparative process of KLT- induced DNA injury, and when the degree of DNA injury is too serious to be repaired by PCNA, other genes such as P53 and Bcl-2 will send signals to trigger the apoptosis of cell and they were also convinced that the anticancer effect of KLT was a result of multiple gene interaction and restriction [8,15,16,19].

c-myc is one of the core protein – myc family in oncogene, and it not only is a positive controlling gene in cellular growth and cell life circle, but also take part in the progress of apoptosis, that is to say, it has dualism[37]. Under a circumstances of growth inhibiting, improper expression of c-myc could induce regulatory failure of normal cell life circle and apoptosis[38]. when studying the KLT- induced the apoptosis of cancer cell(HL60), Li Y make use of RT-PCR to detect the the gene expression of c-myc, she observed that there was no significant change in genetic transcription after 24 hours when using KLT at 10 ul/ml [19]. As bcl-2 gene, we should do further research and study on it.

P21^WAFI/CIPI ^is the downstream gene of p53 gene, its activation contain the p53 dependent path and the p53 non-dependent path[39]. The protein product of P21^WAFI/CIPI ^can combine with cell circle protein, cyclin-dependent kinase(CDK), and proliferating cell nuclear antigen(PCNA) to form a quaternionic complex that can stop the cell life circle and depress the cell growth. Guo JW discovered that while up-regulate the expression of p53 protein, KLT can raise the expression of p21^WAFI/CIPI^mRNA and protein, it indicates that KLT can induce apoptosis of cancer cell by way of the p53 dependent path to up-regulate the expression of p21^WAFI/CIPI ^[16].

Furthermore, some researchers found that KLT can up-regulate the level of ubiquitin C, RAD17 genes and down regulate t the level of cyclin A, cyclin E1, cyclin F gene in studying the influence of KLT on Patu-8988 cell life circle and gene expression [40].

In addition, some study [41] show that the genes regulated apoptosis are not isolated, they can influence and restrict with each other, for example, Bcl-2 family, IAPs family, c-myc, P53, P35, can affect activation of caspase-3 through regulating activation of caspase-8 and caspase-9[42]. so Kanglaite may promote the interaction of those genes to form a network cycle, amplify cascade reaction and further promote apoptosis. but there is no related research at the present stage.

## Conclusion

Now, the study of KLT-induced apoptosis is focused on the above-mentioned gene. In short, KLT induce apoptosis of cancer cell by way of up regulating the expression of p53 gene, Fas gene, Caspase-3, PCNA, p21^WAFI/CIPI ^and down regulating the expression of cyclin A, cyclin E1, cyclin F gene. But its effect on the expression of bcl-2 gene and c-myc gene is not yet clear.

The occurrence of malignant tumor may be caused by the abnormal proliferation of cell or the inhibition of cellular apoptosis pathway. The proliferation of tumor cell and the apoptosis of tumor cell are not only affected by many factors and pathways such as drugs, radioactive ray, etc, but also regulated by some tumor genes or tumor-suppressing gene. It has been proved that the Chinese crude drug-induced apoptosis is one important anticancer mechanism of Chinese crude drug, and it is also relevant to the concentration of the medicine[43]. Just as the result of experimental treatment on hepatoma of mice that we have done [44], we found that KLT can made the cancer cells stop in the G2+M phase of cell life circle, and prevent them form entering the G0 and G1 phase. so it can induce the apoptosis and suppress growth of cells without any effect on the surrounding normal tissues or causing Inflammatory reaction which are unique characteristics. based on these characteristics, KLT has been used in the treatment on many kinds of malignant tumor, and the clinical effect show that KLT can not only repress tumor directly, improve the quality of life obviously, enhance the patient's immunity, but also enhance the effectiveness of chemotherapy and reduce side effects [45-47], which is consistent with its pharmacology above-mentioned However, the relationship between the apoptosis of hepatoma carcinoma cell and gene is further studied, although these research can not fully reveal the mechanism of KLT-induced apoptosis, and its experimental result is still preliminary, the trend of using KLT to prevent and cure liver cancer is already formed, and with the development of modern medical technologies, its mechanism will undoubtedly be revealed in the near future. and KLT will bring new hope for the treatment of cancer and the protection of normal tissues with the more sufficient evidence of its effect on liver cancer and other tumor in clinical trials.

## Competing interests

The authors declare that they have no competing interests.

## Authors' contributions

LY wrote the article under the supervision of DQ. LY, LCS and DQ contributed to the collection and evaluation of date. LY conceived of the study, and participated in its design and coordination. All authors read and approved the final manuscript.
